# Establishing a Diagnostic Autism Assessment Clinic in a Primary Healthcare Setting to Reduce Waiting Times, Increase Efficiencies and to Improve the Patient Journey and Clinical Outcomes

**DOI:** 10.1192/bjo.2022.286

**Published:** 2022-06-20

**Authors:** Nadia Dabbagh, Baraa Dhuhair

**Affiliations:** Rashid Hospital, Dubai Healthy Authority, Dubai, UAE

## Abstract

**Aims:**

To establish a multidisciplinary diagnostic autism assessment clinic in primary healthcare so as to reduce lengthening waiting lists for specialist hospital based services, to increase efficiencies and improve the patient journey and clinical outcome. Timely diagnosis and early access to community based early intervention services optimizes outcomes.

**Methods:**

Waiting time for specialist hospital based services were increasing in number. Analysis of the data revealed that 42.9% of all referrals were autism related and 75.5% of these referrals were for autism assessments in under six-year-olds. Bottlenecks were found in the current system. In collaboration with primary healthcare colleagues, a new pathway was developed with paediatricians, social workers and primary healthcare physicians completing a comprehensive initial assessment including conducting a Childhood Autism Rating Scale (CARS). Each of the three hospital based child psychiatrists then ran a diagnostic autism clinic in the primary healthcare setting once a month (so three clinics in total) to review the initial assessment, meet the child and family/carers and then to confirm the diagnosis and write a medical report for community based services as appropriate. Follow-up care remained in primary healthcare unless there was diagnostic uncertainty, significant behavioural difficulties or comorbidities requiring medication. The project timeline started with one and gradually increased to four diagnostic assessments in each clinic, that is, twelve per month.

**Results:**

57 diagnostic assessments were completed in first eight month period. Waiting times for diagnostic assessments in under six-year-olds were reduced from two to four months to only one to two weeks. Medical reports were issued within five working days. Under six-year-olds and their parents no longer had to attend busy, less child friendly hospital settings but rather were able to attend a purpose build early intervention centre within the primary healthcare setting.

**Conclusion:**

In conclusion, this is an example of a successful quality improvement project embracing the efficiencies of integrated models of care between primary, secondary and tertiary services. Critical success factors included strong leadership support, compelling rationale and purpose, clear clinical pathways and clear roles and responsibilities. It was presented as part of the hospital wide quality meetings in November 2021.

